# Supplementary material for: Are carbon emissions trading and green financial instruments synergistic? -Comprehensive quantitative research based on content analysis
Source: PLoS One. 2024 Mar 7;19(3):e0298601. doi: 10.1371/journal.pone.0298601 (PMC10919601; doi:10.1371/journal.pone.0298601)
Supplement: S1 Data — (ZIP) [file pone.0298601.s001.zip › Data/地级市Z_F工作报告.zip!/╣π╢1⁄2/╔╪╣╪╩╨/└┤╫╘╣1⁄2╓┌║┼í╛┬φ┐╦╩2╛▌═°í┐_2016.docx]

我代表市人民政府，向大会作工作报告，请予审议，并请政协各位委员和其他列席人员提出意见。

一、“十二五”时期和2015年工作回顾

过去五年，市政府在市委的正确领导下，在市人大及其常委会和市政协的监督支持下，全面贯彻落实中央和省委、省政府决策部署，全力推动绿色转型振兴发展，基本完成“十二五”规划的主要目标任务，改革开放和现代化建设取得重大成就。

（一）努力加快经济发展，综合实力迈上新台阶。“十二五”期间，我市地区生产总值突破1000亿元大关，2015年预计达1163亿元，年均增长10%。人均生产总值4万元，年均增长9.5%，居全省山区市首位。来源于韶关的财政总收入突破200亿元、2015年达220亿元，是2010年的1.5倍；2015年地方一般公共预算收入85.2亿元，比2010年增长78.3%。产业结构由2010年的13.7∶42.6∶43.7调整为12.9∶37.5∶49.6，农业增速稳居全省前列，工业产值跃上千亿元台阶。五年累计完成固定资产投资3137亿元、社会消费品零售总额2366亿元、外贸出口总额51亿美元，分别是“十一五”的1.57倍、1.95倍和1.88倍。2015年末金融机构本外币存贷款余额分别为1533亿元和732亿元，是2010年的1.69倍和1.95倍。市场主体突破13万户，比2010年增长34.7%。民营经济稳占半壁江山，成为推动发展的重要力量。

（二）深入推进改革创新，发展环境得到新优化。重点领域改革取得新成效。行政管理体制改革和行政审批制度改革不断深化。完成简政强镇事权改革。乳源完成大部制改革试点。2012年以来累计取消调整审批事项552项。建成网上办事大厅、公共资源交易中心、政务互动平台和12345投诉举报平台。完成公务用车制度改革。农村综合改革、事业单位分类改革以及商事登记、财政、金融、价格、国企、投融资等领域改革深入推进。社会信用体系和市场监管体系建设全面推进。科技、教育、文化、医药卫生体制改革取得新进展。收入分配制度改革和社会治理体制创新稳步推进。创新驱动更加有力，我市荣获“国家科技进步考核先进市”、“产学研结合示范市”称号，专利申请授权量连续十年居全省山区市首位。莞韶对口帮扶成效明显，累计引进落地项目194个，总投资额达422亿元。广韶战略合作关系全面构建，区域合作深化发展。五年累计实际引进资金580亿元、年均增长23%。

（三）狠抓基础设施建设，城乡面貌呈现新变化。以“三大抓手”为重点，全面加快城乡建设。交通基础设施不断完善。五年累计投入318亿元建设交通基础设施，比“十一五”增长18.6%。新增铁路117公里、高速公路327公里，改造国省道743公里、并通过交通运输部“十二五”国省道公路路况检测，完成农村公路硬底化2779公里。韶关电厂“上大压小”项目建成投产，开展大规模电网建设和改造，新增电力装机容量120万千瓦。产业园区逐步成为重要增长极。乳源产业转移工业园纳入省产业转移园管理；仁化县、新丰县依托莞韶园被认定为省产业集聚区，享受省产业转移政策，各获得5000万元专项资金。2015年产业转移园规模以上工业增加值108.6亿元，是2010年的4.8倍；占全市规上工业比重达32.1%，比2010年提高21.1个百分点。芙蓉新区建设全面提速，老城区功能优化提升。五年完成市区市政重点项目投资28亿元，建成第四污水处理厂、工业西片区内涝整治、百年东街、通天塔等一批项目，新铺设截污管网14公里，新增公园绿地面积245公顷。市区建成区由2010年的78平方公里拓展到99.2平方公里，常住人口由79.3万人增加到102.6万人。各县（市）规划建设了新城区或连片改造了旧城，集聚要素能力增强。新农村建设扎实推进。县域经济占全市比重达55%，比2010年提高6.4个百分点。2015年，全市城镇化率达54.8%，比2010年提高2.3个百分点；人均住房面积39平方米、拥有小汽车16.9万辆、宽带用户46万户，分别比2010年增长41%、59%和118%。群众生产生活条件明显改善。

（四）深入推进生态建设，生态文明提升新水平。制定实施主体功能区规划。乐昌、南雄、始兴、仁化、乳源被纳入国家重点生态功能区。我市在省实行最严格水资源管理制度考核中获得优秀等次。跻身省林业生态市和国家生态文明先行示范区行列。石漠化治理成效明显，城乡生态环境改善。五年累计造林204.5万亩，建成生态景观林带473.5公里，“三边”造林9.5万亩，有林地面积净增82.7万亩，生态公益林面积扩大290万亩，森林覆盖率提高3.5个百分点、达74.95%。利用列入国家节能减排财政政策综合示范城市的机遇，加强综合防治，全面完成省下达的节能减排任务。五年淘汰落后水泥产能508万吨、钢铁75万吨、蓄电池17.5万千伏安时、造纸4.2万吨，减排二氧化硫1.9万吨。城镇生活污水集中处理率达75%以上。规模以上工业增加值综合能耗和万元生产总值能耗分别比2010年下降44.3%、22.8%。加强大气、水、噪音污染防治，开展对重点工矿企业重金属污染综合整治和环境修复。推广新能源汽车804辆，淘汰“黄标车”及老旧汽车1.94万辆。五年完成1157项污染减排工程。综合整治农村人居环境，乡村“清洁美工程”成效明显。2015年空气质量优良率达94.2%。通过城乡建设用地增减挂钩、“三旧”改造、严打违法非法行为等综合措施，促进土地节约集约利用。

（五）持续加强民生事业，社会建设取得新进步。2015年民生支出占财政支出比重达80.6%，比2010年提高18.2个百分点。城镇居民人均可支配收入2.34万元，农村居民人均可支配收入1.15万元，分别比2010年增长68.3%和82.5%。城镇居民恩格尔系数下降到37.7%。市区居民消费价格指数涨幅为1.2%。五年累计新增城镇就业26.5万人，培训农村劳动力11.2万人，转移就业26.1万人。城镇登记失业率控制在3%以内。实施城乡居民大病保险，基本实现社会保险全覆盖。底线民生保障水平明显提高。完成第三次全国文物普查和各项文化惠民工程任务。19个项目入选国家级或省级非物质文化遗产名录。基层医疗卫生和公共卫生服务体系不断完善，人口和计划生育工作稳步推进。建成95个乡镇农民体育健身工程，免费向社会开放55万平方米体育场馆，举办了市第十四届运动会。社会管理综合治理全面加强，安全生产“四项指数”全面下降，食品药品安全监管水平提高，信访形势保持平稳，“平安韶关”创建成效明显。国防建设和双拥工作有新成绩。妇女儿童、残疾人、对台、民族宗教、外事侨务、司法、史志、统计、审计、粮食、档案、供销、新闻出版、气象水文、防震减灾、人防应急等工作取得新进步。

（六）全力办好一批大事，城市形象实现新提升。“十二五”时期，我们着力办了一批既利当前又利长远的大事。我们全力推进教育创强争先，率先在全省山区市普及15年基础教育，实现省教育强县（市、区）和国家义务教育发展基本均衡县（市、区）全覆盖。我们全力推进城乡防灾减灾体系建设，基本完成山区中小河流治理550公里，建成乐昌峡水利枢纽，市区防洪标准大幅提高。我们全力打好高速公路建设大会战，南韶、乐广、大广高速公路建成通车，实现县县通高速公路，成为粤北首个、全省第四个拥有环城高速公路的城市。我们全力推进棚户区改造，成为全省棚户区改造主战场，建成和基本建成2.41万套，基本建成原曲仁矿棚户区改造这个全省最大的棚改项目，在主城区东部初步形成一个城市新组团。我们举全市之力建设芙蓉新区，起步区五年累计投资约96亿元，骨干道路联网成环，城市功能不断完善，产业集聚初见成效，人气明显提升，城市总体框架基本成型。我们深入推进扶贫开发“双到”工作，五年累计落实帮扶资金39.9亿元，改建低收入住房困难户住房2.53万户，搬迁“两不具备”贫困村庄277个。我们成功承办广东国际旅游文化节、禅宗六祖文化节、2015年中国佛教讲经交流会，成功创建第九届全国双拥模范城，成为全省首批“全国文明城市提名资格”地级市。这些大事，提升了韶关美誉度和知名度，增强了城市凝聚力和影响力。

（七）大力推进依法行政，政府建设开创新局面。我市成为全省首批被授予地方立法权的设区市。坚持向市人大及其常委会报告工作，向市政协通报情况，自觉接受监督。虚心听取各民主党派、工商联、人民团体、无党派人士和社会各界的意见建议，加强与工会、共青团、妇联等人民团体的联系。五年累计办理市人大议案1件、代表建议368件，市政协建议案18件、提案722件。在交通运输、城市管理等领域推行综合执法，行政执法监督加强。建立重大行政决策专家咨询论证、风险评估等制度，规范决策程序，政府决策水平进一步提高。实现了村（社区）法律顾问全覆盖，“六五”普法全面完成。行政复议和政府法律顾问工作加强。建成政府信息公开平台，政务公开深化发展。深入开展党的群众路线教育实践活动和“三严三实”专题教育，认真落实中央八项规定精神，坚决整治“四风”，“三公”经费大幅下降。机关效能建设、廉政建设和纠风治理工作取得实效。

2015年是“十二五”规划的收官之年。面对错综复杂的发展环境和经济下行压力持续加大的严峻形势，我们沉着应对，迎难而上，扎实推动经济运行企稳向好，全面冲刺“十二五”规划目标，努力为“十三五”的发展奠定更加坚实的基础。一是全力稳增长。坚持问题和目标导向，多措并举，着力推进“三大抓手”，大力促进消费，支持实体经济发展。全市生产总值由一季度的3.8%回升到全年的6.5%左右。地方一般公共预算收入增长4%，社会消费品零售总额增长10.8%。旅游接待人次、旅游收入分别增长13%和20%。二是大力促合作。莞韶对口帮扶深化发展，融入珠三角扎实有效。我市分别与广州市政府以及前海人寿、华侨城、中信正业、保利文化、广州港等企业签订合作框架协议。省政府批准建设珠江西岸先进装备制造产业带韶关配套区，支持打造“广佛肇+清云韶”经济圈。全市新签内联项目620个，投资总额266亿元，实际到位289亿元。三是强力优环境。建立招商引资工作联席会议制度。出台促进民营经济发展49条、招商引资19条、发展大旅游21条、进一步优化投资营商环境36条和加强督查督办推进落实提高执行力等一揽子政策措施，开展不作为、慢作为、乱作为整治。对小微企业和个体工商户免征42项行政事业性收费。四是着力增活力。公布市直部门权责清单和职能调整目录。全面取消“非行政许可审批”类别。实施“三证合一、一照一码”商事登记制度改革，市场主体增长8.9%。稳步放开取消下放62项政府定价项目。优化国有资本配置，组建市金财投资集团。设立熙正产业基金、旅游产业发展基金、众创基金和中小微企业发展基金，健全政策性担保体系，着力缓解企业融资难。引进战略投资者参与国企改革。首次举办PPP项目推介会。积极开展县镇村三级政务服务平台试点，乳源经验在全省推广。五是努力惠民生。市政府公开承诺办好的九件实事基本完成。全市民生支出完成226亿元，增长58%。基本公共服务均等化水平进一步提高。新开工各类保障性住房2.71万套，基本建成1.58万套，超额完成任务。完成576公里新农村公路路面硬底化。完成市区11万户自来水“一户一表”改造。

各位代表，过去五年的成绩来之不易，这是全市上下团结奋斗的结果。在此，我代表韶关市人民政府，向全市人民、驻韶部队官兵、各民主党派、人民团体以及社会各界人士表示崇高的敬意！向所有关心和支持韶关振兴发展的港澳同胞、台湾同胞、海外侨胞和国际友人表示衷心的感谢！

回顾过去五年的工作，我们深刻体会到，加快新常态下的发展，一是必须抢抓发展机遇，坚持发展第一要务，解放思想，增强忧患意识、责任意识和发展意识，主动作为，顺势而为，奋发有为，聚精会神谋发展，凝心聚力抓落实；二是必须扩大有效投资，创新理念抓项目，突出重点抓项目，注重实效抓项目，大抓项目抓大项目，以有效投入保增长、调结构、促转型；三是必须扩量提质发展，加大招商引资力度，正确处理量与质的关系，优化存量与培育增量并举，做大总量与提升质量并重，推动产业结构优化升级，加快实现产业兴韶；四是必须生态优先发展，正确处理发展与保护的关系，巩固生态屏障功能，促进绿色低碳循环发展，走生态文明发展道路；五是必须优化发展环境，提高政府效能，加强社会建设，改善人居环境，营造法治化国际化营商环境；六是必须深化改革开放，积极走出去请进来，主动融入珠三角加快发展，与时俱进创新体制机制，充分激发市场活力和社会创造力；七是必须发展惠民利民，坚守底线民生、保障基本民生、关注热点民生，使广大群众在共建共享的发展中增强获得感，凝聚发展正能量，增强发展新动力；八是必须狠抓工作落实，紧盯目标任务，强化责任担当、紧抓快办、扭住不放、一抓到底，确保市委市政府各项决策部署不折不扣落实到位。

在看到成绩的同时，我们也清醒认识到，我市发展仍面临不少困难和问题，发展不足仍然是主要矛盾，我市发展长期积累的经济结构不合理、创新驱动乏力、内生动力不强、市场活力不足的问题仍然突出。特别是在适应新常态、应对经济下行的严峻形势时，问题进一步凸显：经济产业结构单一、企业所有制结构单一的历史性困局仍没破解，发展方式粗放，转型升级任务艰巨；重点项目储备不足，投资拉动作用减弱，发展后劲不强；资源环境约束加剧，节能减排形势严峻；社会事业历史欠账多，公共服务任务艰巨。同时，改革任务繁重，政府职能转变还不到位，责任意识和服务意识有待增强，执行力有待提高。对此，我们将高度重视，采取有效措施认真加以解决。

二、“十三五”时期奋斗目标和主要任务

“十三五”时期，是我市战略转型产业兴韶、与全国同步全面建成小康社会的攻坚阶段，也是我们大有可为的重要战略机遇期。机遇与挑战并存，合作与竞争同在。我们必须认清形势，增强忧患意识、责任意识，抢抓机遇，奋发进取，有效应对风险和挑战，努力掌握主动权，不断开拓发展新境界。

“十三五”时期政府工作的总体要求和主要目标是：高举中国特色社会主义伟大旗帜，以马克思列宁主义、毛泽东思想、邓小平理论、“三个代表”重要思想和科学发展观为指导，全面贯彻党的十八大和十八届三中、四中、五中全会以及省委十一届二次、三次、四次、五次全会精神，深入贯彻习近平总书记系列重要讲话精神，按照“四个全面”战略布局，坚持发展第一要务，坚持创新、协调、绿色、开放、共享发展理念，坚持全面深化改革，稳中求进，统筹推进“五位一体”总体布局，着力提高质量效益，努力将韶关建设成为珠三角融合发展区、国家生态文明先行示范区、“一带一路”节点城市，确保与全国同步全面建成小康社会。“十三五”时期的主要目标是“一个同步，四个基本”：与全国同步全面建成小康社会；基本建立与珠三角地区融合发展的新格局，基本建立具有区域竞争力的现代产业新体系，基本建成功能完善的现代化新型城市，基本形成有利于加快发展的新机制。为实现上述目标，要认真实施“一一二三八”发展方略。

瞄准一个总目标，即与全国同步全面建成小康社会。2020年全市生产总值达1800亿元（2010年不变价）；人均生产总值6万元；城乡居民人均收入达2.5万元；小康指数达到97%，广大群众幸福感普遍提升。强化创新驱动，深化改革开放，促进城乡区域协调发展，促进新型工业化、信息化、城镇化、农业现代化同步发展，促进经济与社会、物质文明与精神文明协调发展。建设绿色美丽韶关。打赢扶贫开发攻坚战，加强公共服务，创新社会治理。

坚持一个总战略，即主动融入珠三角。建立健全区域合作机制，实现与珠三角互补发展、互利共赢。推进“广佛肇＋清云韶”经济圈一体化建设合作机制取得重大突破，在交通、产业、市场、人才等领域对接合作实现重大突破。建设珠江西岸先进装备制造产业带韶关配套区、珠三角生态休闲区和珠三角连接内陆腹地桥头堡。

突出两个主战场，即城市经济和县域经济。发挥各县（市、区）比较优势，构建区域错位发展、协调发展、共赢发展新格局。打造市辖三区、乳源东北部和乐昌、南雄城区“3＋1＋2”城市经济圈；乐昌、南雄建成为粤北门户城市，新丰、翁源、乳源建成为主动融入珠三角先行区，仁化、始兴建成为生态休闲示范区。

强化“三大抓手”。充分发挥交通基础设施的先导作用、产业建设的支撑作用、城市建设的承载作用，推动发展新突破。加快交通基础设施建设，构建“八高四铁二航”的现代综合交通运输体系。健全产业转移区域合作机制，加快“一园一城七组团”建设；打造自贸区大宗商品仓储基地和保税物流配套区，促进产业园区扩能增效。坚持“五个融合”和“五个优先”，全面加快芙蓉新区建设，打造北江明珠；深入推进老城区提质优化，加速曲江同城一体化，促进中心城区扩容提质。

大力发展八大产业。实施产业发展三年行动计划，加快发展战略性新兴产业，依靠改革创新扩增量、优存量、促转型，重点加快发展八大战略性支柱产业，着力打造若干个产值500亿元以上的支柱产业，加快实现产业兴韶。大力发展钢铁深加工产业，建成全省重要的金属原材料供应基地；大力发展先进装备制造业，初步建成广东重要的装备制造业基地和珠江西岸先进装备制造产业带韶关配套区；大力发展能源电力产业，建成全省重要电力能源生产输出基地；大力发展大旅游，建成广东生态旅游休闲区和国家旅游产业集聚区；大力发展大物流，打造珠三角大宗商品仓储物流基地和粤湘赣商贸物流基地；大力发展大农业，建成特色农产品主产区、港澳和珠三角农产品供应地；大力发展新材料产业，打造各具特色的新材料产业园区（基地）；大力发展特色轻工业，打造轻工业特色区域和产业集群。同时，积极改造提升其他传统优势产业，加强与珠三角地区的产业协作，推动传统产业新一轮技术改造，拓展产业链，提升价值链。大力发展港口经济，积极打造高铁经济、航道经济、航空经济和高速公路经济，推动港口、城市、产业的联动发展和集群发展。

三、2016年工作安排

2016年是“十三五”的“打基础、抓落实”之年，也是在新起点上全面融入珠三角加快发展的关键之年。今年政府工作的总体要求是：全面贯彻党的十八大和十八届三中、四中、五中全会及中央经济工作会议精神，深入贯彻习近平总书记系列重要讲话精神，按照省委、省政府和市委的部署，以全面融入“广佛肇＋清云韶”经济圈为契机，以提高质量效益为中心，聚精会神谋发展、凝心聚力抓落实，全力推进战略转型产业兴韶，加快振兴发展，为实现“十三五”规划目标打好基础。

全市经济社会发展的主要预期目标是：生产总值增长10%左右；人均生产总值增长9%左右；固定资产投资增长15%；社会消费品零售总额增长11%；地方一般公共预算收入增长5%；城乡居民人均可支配收入增长9%；城镇登记失业率控制在3.5%以内；居民消费价格指数涨幅控制在3%左右；研究与发展经费支出占地区生产总值比例提高到1.3%；完成省下达的节能减排等任务。

围绕上述目标，今年重点抓好以下八个方面的工作：

（一）着力强化投资拉动，促进发展提速。

加强基础设施建设。完成韶关丹霞山机场项目前期工作，争取年内动工建设；完成机场空港产业园规划编制，启动空港基础设施规划建设，加快打造区域航空快递物流中心和通用航空服务基地，促进空港经济发展。加快推进武深高速、汕昆高速、韶新高速、芙蓉新城客运枢纽和东郊客运枢纽等项目建设。建成武广高铁乐昌东站。完成100公里以上国省道干线改造和300公里农村公路建设。加快北江航道扩能升级和韶关新港、韶关港水运口岸等项目建设。实施“光网城市”工程，加快城市光纤入户和农村宽带网络建设，推动城乡基础设施智能化。加快市电子政务云平台、广州超算中心韶关分中心建设。加快韶关电网改造和国粤韶关综合利用发电、华电南雄热电联产、韶能新丰生物质发电等项目建设。加快推进南水水库供水工程建设。完成市区小岛片区旧堤加固工程。完成2015年度高标准基本农田建设23.63万亩。抓好小农水重点县、中小型灌区改造等民生水利工程建设。

加强招商引资。更新招商理念，改革招商体制，创新招商模式，发挥投资人、专家学者、中介组织的招商积极性，力争在引进先进装备制造业方面取得新突破。实行招商引资“一把手”工程，完善招商引资项目库、乡贤信息库。围绕重点产业补链强链、招大引强，引进战略投资者和行业大中型骨干企业20家以上。落实“四个一”和“四定”工作机制，用好乳源少数民族地区政策，优化投资营商环境，推动项目落地开工和企业投产达效。力争新签约项目总投资250亿元以上、新签约项目动工率50%以上、实际利用外资增长5%以上。深入研究珠三角产业体系，重点围绕珠江西岸先进装备制造业和深莞惠电子信息业，有针对性开展产业链招商。完善莞韶华南钢铁深加工科技产业园的规划编制，做好土地调规工作，完成5000亩土地征拆和基础设施建设；积极开展面向珠三角的产业招商，加快推进宝钢（韶钢）集团在韶关建设优特钢长材精品基地，建设机器人特种金属材料生产基地，打造集钢铁深加工、汽车零部件、整机生产和产研结合的珠江西岸先进装备制造产业带核心配套区。力争特钢园新引进项目总投资20亿元，到位资金10亿元。

撬动社会资本投资。整合优化财政资金和国有资源，充分发挥财政资金和国有资本的杠杆作用，精准发力，引导民间扩大投资。制定民间资本投资准入负面清单，放宽市场准入，鼓励进入交通基础设施、社会事业、保障性安居工程、生态环保等领域。支持战略投资者和民营资本参与国企改革。推动投融资模式创新，建立健全PPP项目库，积极开展政府与社会资本合作。

加强项目要素保障。深入研究政策，着力解决项目征拆、土地报批、资金筹措、环评、行政审批等瓶颈问题。完善市领导联系重点项目制度，加强重点项目调度。完善政府投资项目审核运行、重大项目绿色审批通道、跟踪协调机制，推动项目早开工早竣工。落实政府投资项目计划，推动政府投资规范化制度化。突出抓好51个市重点建设项目，完成投资253.8亿元以上。做深做实重大项目前期工作，建立健全重点项目库和重点项目储备库。加强沟通对接，争取更多项目纳入国家和省的盘子。

（二）着力加强产业培育，壮大实体经济。

推进工业转型攻坚。加强莞韶城、粤商高科创新园、特钢园等工业平台建设，积极申报国家级高新技术开发区，加强北部产业园规划，支持翁源经济开发区申报享受省级产业园政策。深入实施工业转型升级攻坚战三年行动计划，重点推动钢铁、冶金行业与珠三角产业对接，建立稳定协作关系。推进珠江西岸先进装备制造产业带韶关配套区建设，提升产业发展水平，打造粤北汽车及零部件生产基地和矿山建筑工程机械研发生产基地。落实市领导联系重点企业制度，深化“一企一策”点对点服务，重点推进都市丽人、新丰稀土分离厂、南雄彤置富水泥、台泥（韶关）水泥生产配套生活垃圾处理等项目建设。狠抓新一轮技术改造，落实省智能制造发展规划和机器人发展专项行动计划，促进韶钢改造、韶烟二期技改、大宝山铜硫资源综合利用示范基地等一批重大技改项目加快投产达效，完成技改投资84亿元。编制工业新增长点目录，新增规模以上工业企业30户，实现工业增加值增长11.6%。

加快服务业发展。实施好旅游产业三年行动计划，用好旅游发展专项资金和旅游交通公路建设引导基金，全力推动全域旅游发展。改革丹霞山景区管理体制，强化与华侨城、保利文化等战略投资者的合作，加快环丹霞山旅游产业园建设。推动大南华文化旅游创意产业园和马坝人遗址公园建设。制定乡村旅游发展指导意见和乡村民宿、乡村旅游驿站建设标准，完善乡村旅游配套设施。推出“九龄故里·百里画廊”等一批精品旅游示范线路。创建全国休闲农业和乡村旅游示范项目。推进“互联网+旅游”，建设韶关智慧旅游公共管理和服务信息平台。健全旅游联合营销机制，打响旅游节庆品牌。做好“旅游+”文章，培育新业态新产品，形成消费新热点。加快发展商贸流通业。推进贸易本地化，鼓励制造企业扩大物流外包需求，鼓励广晟、韶钢和粤电集团在韶设立物流法人机构。支持社会资本建设大型专业批发市场和商贸流通企业，重点推进鑫金汇、新雪域、粤北农特产品电商物流商贸城等项目建设。加快黄沙坪创新园和市电子商务产业园建设，规划建设快递产业园。大力发展跨境电商，重点建设广东邮政EMS国际快件分拨中心。

大力发展特色农业。落实强农惠农富农政策，推进一二三产业融合发展。划定永久基本农田保护红线。着力保障粮食安全和农产品质量安全。加强农业科技推广。推进农村土地流转，发展适度规模经营。因地制宜发展特色种养业、加工业，培育专业村、专业镇，推进农业产业化、标准化、品牌化、信息化。加快国家现代农业示范区、粤台农业合作试验区等农业园区建设，壮大设施农业、生态农业和休闲观光农业。大力培育新型职业农民，发展壮大多元农业经营主体。大力发展特色林业经济，打造南岭茶叶产业带，建设中药材种植基地。加强农产品流通设施和市场建设。扶持发展农村电子商务，培育“互联网+农业”新业态，打造电商创业村，促进农产品精深加工和农村服务业发展。

（三）着力强化双创支撑，增强创新能力。

实施“互联网+”行动计划。鼓励企业实施智能制造提升工程，抓好韶钢、东阳光等企业“两化融合”管理体系贯标试点。支持韶乐购、U购网等电商发展和南雄创建全国电子商务进农村综合示范市，打造省级电子商务试点示范城市。推动发展“互联网+产业”等项目，促进互联网与经济社会各领域深度融合。

鼓励大众创业万众创新。大力培育科技企业孵化器，加强孵化育成服务，发展众创空间。完善评估机制和创新创业激励政策，扶持东莞韶关众创平台、黄沙坪创新园、大学生创业孵化基地建设，力争全市新登记初创企业户数增长10%以上。实施创业培训补贴，扶持初创企业发展。支持科技企业孵化器拟毕业企业入驻园区扩大生产。落实加快创新驱动发展“1+N”政策，大力发展高新技术产业，加快科技成果转化。支持组建产业技术创新联盟，大力发展新型研发机构，推动高新技术企业、研发机构落户韶关。

加快发展民营经济。加快小企业创业基地建设。用好产业、创投等基金和“助保贷”、“过桥贷”，完善中小微企业信贷风险补偿机制和担保体系。支持中小微企业进入股权、债券等市场直接融资。支持民营企业参与PPP项目和国有、集体企业改制重组。搭建信息服务、技术支撑、公共检测、“互联网+”营销、“产学研”应用合作和企业素质提升等六大服务平台，推进质量检验检测技术平台建设。鼓励个转企、企转规、规改股、股上市，支持民营企业转型升级、做强做大。

加强人才队伍建设。加大人才引进培养力度，完善高层次人才信息库和服务体系，优化人才政策，着力营造尊重人才、鼓励创造、激励创新的氛围。加强新型智库建设。大力引进、用好创新创业领军团队和人才。落实国家中小企业银河培训工程，实施企业家和高端技术人才培养工程。加强高技能培训基地实训项目和技能大师工作室建设，推进职业教育资源整合，着力加强高技能人才队伍培育。支持韶关学院内涵发展，鼓励职业院校与企业建立紧密用工协作关系，支持校企合作办学。

（四）着力扩容提质，加快城乡发展。

强化规划引领。修编城市总体规划和城区综合交通规划，完善城市近期建设规划。积极谋划芙蓉新城起步区南片区项目规划和城市设计，编制曲江新城规划，完成小岛核心区风貌整治规划、城市防涝（雨水）详细规划编制。推进美丽（特色）村庄、市级中心镇和古村落等镇村规划编制，提高规划覆盖率。加强各专项规划及城市总体规划的协同，推进“多规合一”，加大控制性详细规划的编制和规划信息化工作力度，加强规划公开性强制性。

加强城市建设。落实芙蓉新城三年基本成城行动方案，加快新区建设。全面完成农民安置新村建设，抓好拆旧工作。推进前海人寿金融中心、中海投资、人保财险、农信社、保利商业综合体等总部经济项目建设。加快滨江公园、华师附中等公建项目建设。加快芙蓉隧道、莲花大道（东环线）二期、江湾大桥、滨江路、曲江大道、新白线等道路建设。推进老城区提质优化，完成韶关大道、芙蓉北路等主次干道以及主道路信号灯升级改造，整治城市内涝和交通拥堵，完成城乡结合部路灯安装，新建一批市民健身广场。稳妥谋划有轨快速公交，统筹开发公交线路，完善新能源汽车配套设施。合理规划建设公共停车场，扩大停车场建设配比，缓解市区停车难问题。实现市区和县城周边镇镇通公交车。推进城市地下综合管廊建设，完成市区地下管线普查，筹建管道运营公司。促进房地产市场平稳健康发展。支持各县（市）加快城市新区和重点中心镇建设，推进基础设施网络化，提高集聚能力。抓好省级新型城镇化建设试点工作。

加强城乡管理。强化“数字城管”建设，打造智慧城市，提高城市管理水平。健全环境卫生、交通秩序整治和城市内涝综合防治等长效机制，促进城镇管理常态化、规范化、精细化。统筹农村村村通自来水工程、改水改厕、垃圾处理等工作，综合整治农村人居环境。制定污水、垃圾处理设施全覆盖规划，抓好铁路、高速公路和国省道公路沿线环境整治，实现村庄保洁覆盖面达70%、农村生活垃圾处理率达75%以上。抓好村级公益事业建设“一事一议”项目和五彩瑶乡、丹霞彩虹、“千年驿道、广府原乡”等新农村连片示范项目建设。加强历史文化名村、历史建筑、传统村落、古树名木保护。加强城乡规划管理，深化“两违”综合治理。

（五）着力深化改革开放，激发市场活力。

推进重点领域改革。抓好权责清单组织实施，完善市级行政许可事项保留目录，落实行政许可监督检查和年度报告，全面推进市、县两级政府行政审批标准化。加强供给侧结构性改革，着力去产能、去库存、去杠杆、降成本、补短板。深化投融资体制改革，对企业投资项目实现“三单”管理。理顺市与市辖区权责关系，力争在城市管理、国土资源、城乡规划、环境保护等方面取得突破。深化城市管理体制改革。扎实推进户籍制度改革，全面实行城乡统一户口登记制度。推进不动产登记制度改革。深化事业单位分类改革。继续深化商事登记制度改革，加强后续监管。健全财政转移支付制度，完善财政保障基本公共服务供给机制，深化政府购买服务。深化国企改革，改革市属企业负责人薪酬制度。深化农村综合改革，完成农村土地承包经营权确权登记颁证工作。推进农村集体“三资”管理平台和基层公共服务综合平台建设。加快推进林地确权登记发证到户，抓好国有林场改革。

全面拓展区域协作。实施主动融入珠三角实现加快发展行动计划。依托“广佛肇+清云韶”经济圈沟通协作机制，加强重大合作项目建设，实现协同发展。建立健全广韶两市紧密合作机制，突出围绕交通、产业、工业、旅游、金融、物流、环保和社会公共事业合作，谋划落实一批具体合作项目。巩固和深化莞韶对口帮扶工作，建立对口帮扶重大项目管理台帐，实施对口帮扶评估考核和创新工作评选。加强与红三角城市合作，推进粤湘开放合作试验区建设。

推动金融服务创新发展。大力发展产业金融、绿色金融、科技金融和普惠金融，补齐金融短板。优化金融生态环境，鼓励引导银行业扩大信贷投放。探索建立政策性再担保公司和开展小额贷款保证保险业务，实施“政银保”金融服务项目。大力发展多层次资本市场，积极培育企业上市，完善上市后备企业库，推动3－5家优质企业上市融资。鼓励企业进入新三板及区域股权市场挂牌融资。发展融资租赁和金融租赁。支持城投公司发行企业债券。加强与基金合作，吸引金融机构来韶设立分支机构。引导和支持P2P、众筹等新型互联网金融企业在韶发展。推进普惠金融村村通和农信社改制，支持设立新型农村金融机构，建设社区金融服务站。创新中小微企业、农村特别是贫困地区的金融服务。

大力发展对外经贸。推广自贸区改革试点经验，主动对接广州南沙自贸试验区，设立韶关跨境电商体验店。用好扶持外贸发展政策，促进外贸优进选出。推广旅游购物出口。强化出口订单信保和融资支持，鼓励企业有效扩大出口规模。积极参与“一带一路”建设，支持企业设立境外营销网点。积极利用外资，拓宽利用外资领域，鼓励外资企业增资扩股。加快韶关无水港建设，做强做优“铁海联运”，提高通关便利化水平。实现外贸进出口增长2%。

（六）着力抓好生态环保，建设美丽韶关。

加强生态建设。推进国家生态文明先行示范区和主体功能区建设。抓好森林进城围城、生态景观林带、碳汇造林、乡村绿化美化等林业重点生态工程建设，完成造林28.8万亩，新增29个森林（湿地）公园。划定生态红线，扩大生态公益林面积，提高林分质量。加强封山育林。严格林木采伐管理。加快森林防火远程视频监控系统和航空护林基地建设，加强自然保护区、森林（湿地）公园等生态重要地区森林资源管护。加快南岭生态气象中心建设。

强化环境保护。大力发展循环经济、推行清洁生产，实行污染物排放总量控制。落实大气、水、土壤污染防治行动计划。突出大气污染防治，加强工业废气、施工扬尘、油烟、机动车尾气的治理，淘汰“黄标车”和综合治理老旧汽车，严管秸秆、生活垃圾和建筑废弃物露天焚烧。继续推广新能源汽车，推进碳排放权交易、碳普惠制试点工作。加快污水处理设施建设，城区污水处理率达80%以上。抓好规模化畜禽养殖场治理。落实最严格的水资源管理制度，开展省水生态文明城市建设试点。强化土壤污染防治，对重金属、化学品生产和危险固废处理等重点企业，实行全过程监管和风险防控，严打违法非法行为。抓好水土流失、尾矿库、石漠化等生态脆弱区治理。

节约集约利用资源。编制2016－2025年土地储备规划。用好国家支持城市更新、棚户区改造等政策，盘活存量土地，推进节约集约用地。鼓励和引导企业建设和使用多层标准厂房。完善土地供后监管机制，建立闲置用地清查督导机制。全面推进土地信息化管理工作。探索推行合同能源管理和合同节水管理。严格耕地保护，加强耕地占补平衡审查和耕地储备指标管理。大力倡导绿色低碳发展方式和生活方式。

（七）着力保障改善民生，推进共享发展。

加大精准扶贫精准脱贫力度。启动第三轮扶贫开发“双到”工作。统筹协调扶贫规划与城镇化规划，整合扶贫资金资源，加强贫困村交通、水利、电力、信息等基础设施建设，加大产业、智力、科技和电商扶贫力度。完成4541户农村低收入住房困难户危房改造。通过输出劳务、发展产业、加强培训、推动创业，因人因地施策，促进有劳动能力的贫困户脱贫，对无劳动能力的实行政策兜底，以精准帮扶促进贫困村贫困户精准脱贫。

增强社会保障能力。实施积极的就业创业政策，统筹做好大中专毕业生、农村转移劳动力、城镇困难人员、退役军人就业工作。扩大社会保险覆盖面，推进机关事业单位养老保险制度改革，建立健全职业年金制度。抓好被征地农民社保工作。完善城乡居民大病保险和城镇职工基本医疗保险政策，出台重特大疾病特殊医保补助政策。推进建筑业工伤保险，落实农房险等政策性保险，开展计生家庭意外险等三农保险。完善医保付费方式，实现省内异地就医即时结算。出台政策鼓励社会资本进入养老服务领域，推进医疗卫生与养老服务融合发展。开展“银龄安康行动”。加强保障性住房建设和管理，推进公租房保障货币化，新开工各类保障性住房3200套以上，基本建成3700套。

大力发展社会事业。加强社会主义核心价值观宣传教育，打造一批主题公园（广场）。进一步推进各类教育均衡优质发展，推进市区基础教育资源整合，创建广东推进教育现代化先进市。落实文化惠民工程任务，推进基层文体中心和基层公共数字文化服务管理平台建设。加快建设市民文化活动中心等标志性文化场馆。加大文化遗产保护力度。出台政策鼓励和引导社会力量参与提供公共文化服务。加快文化产业发展。加强创文申名工作。深化医药卫生体制改革，落实分级诊疗制度，推进医疗资源共享和下沉，逐步实现大病不出县，实现社区卫生服务中心全覆盖。加快中医药发展。加强流动人口计生服务管理，全面落实二孩政策。广泛开展全民健身活动，完善体育设施，发展体育产业。完善食品安全“黑名单”制度和突发事件预警信息发布体系。

加强社会治理创新。加强市场监管体系和社会信用体系建设。落实安全生产主体责任，坚决遏制重特大事故发生。推进社会管理重心下移，积极探索“城中村”、城乡结合部、老城区居民区综合治理模式，逐步推行社区化管理。加强社会组织管理，推进社会组织诚信和行业自律。加强矛盾纠纷排查调处，积极化解信访积案。深入开展双拥共建，推进军民融合深度发展，积极支持国防和军队改革。推进和谐劳动关系示范区创建。全面建成县镇村三级公共法律服务体系实体平台。加快公安“四项建设”，完善社会治安防控体系，大力创建“平安韶关”。开展老旧小区物业管理规范化行动，加强物业和快递行业管理。

今年，市政府重点办好十件民生实事：1.完成市区主次干道8个内涝点整治；2.完成山区中小河流治理625公里；3.实施老城区提质便民工程；4.建设韶关教育云数据中心；5.建设百所中小学校（幼儿园）示范性平安食堂；6.加大对困难弱势群体帮扶力度；7.强化低收入住房困难群体住房保障；8.改善农村生产生活条件；9.促进创业带动就业；10.解决浈江区、武江区30个社区公共服务用房。

（八）着力推进依法行政，建设法治政府。

坚持做到依法行政。自觉接受人大及其常委会和人民政协、新闻舆论、人民群众的监督，认真办理人大代表议案、建议和政协委员提案，主动听取社会各界的意见建议。强化依法行政意识，落实市政府常务会议学法制度，加强政府立法和规范性文件制度建设。落实重大行政决策法定程序，加强重大决策公众参与平台建设。完善政府法律顾问制度。深化行政执法体制改革，加强行政执法检查，规范行政执法行为。深化政务公开。加强行政复议和行政应诉工作，完善行政争议协调化解机制。

坚持做到高效务实。始终牢记政治责任，把纪律建设摆在更加突出位置，严守政治纪律和政治规矩。弘扬“三严三实”作风，加强公务员队伍建设，全面提升政府工作人员服务水平。加快行政服务中心功能升级，实现“两集中两到位”，探索将部门分设窗口整合为综合窗口，建设“一门式”政务服务体系。推动实体政务大厅向网上办事大厅延伸，推进“互联网+公共服务”。试行简单事项立等审批、联办事项一口办理、关联事项一章多效、网上审批一次领证、踏勘验收统一勘验的审批流程，方便群众办事创业。加强现场办公和现场督办，建立科学高效的督查督办制度。加大问责追责力度，建立健全问责追责制度，大力整治“慢浮僵推梗懒怕”七种问题，坚决纠正不作为、乱作为。建立健全窗口服务投诉处理机制，加强12345投诉举报平台建设。打造“东莞速度+韶关服务”品牌。

坚持做到清正廉洁。严格落实中央八项规定精神和“两个责任”实体制度，深化标本兼治，加强廉政建设。健全惩治和预防腐败体系，从源头上预防和治理腐败。强化对权力运行的制约和监督，突出工程建设、矿产开发、土地出让和国有资产管理等重点领域，推进廉政风险防控工作全覆盖。加大行政监察和审计监督力度，严肃查处各类违纪违法案件。切实纠正部门和行业的不正之风，着力解决损害群众利益的突出问题。努力做到干部清正、政府清廉、政治清明，不断改善政治生态。

各位代表，“十三五”时期的宏伟蓝图已经绘就，形势催人奋进，发展任重道远！做好2016年的工作，任务艰巨、责任重大、使命光荣。让我们在市委的正确领导下，以更加开放的思想，更加昂扬的斗志，更加扎实的工作，为跟上全省科学发展步伐、与全国同步全面建成小康社会而努力奋斗！
